# Age- and cause-specific contributions to the life expectancy gap between Medical Aid recipients and National Health Insurance beneficiaries in Korea, 2008–2017

**DOI:** 10.1371/journal.pone.0241755

**Published:** 2020-11-03

**Authors:** Jinwook Bahk, Hee-Yeon Kang, Young-Ho Khang

**Affiliations:** 1 Department of Public Health, Keimyung University, Daegu, South Korea; 2 Department of Health Policy and Management, Seoul National University College of Medicine, Seoul, South Korea; 3 Institute of Health Policy and Management, Seoul National University Medical Research Center, Seoul, South Korea; University of Utah, UNITED STATES

## Abstract

Recipients of Medical Aid, a government-funded social assistance program for the poor, have a shorter life expectancy than National Health Insurance beneficiaries in Korea. This study aims to explore the contributions of age and major causes of death to the life expectancy difference between the two groups. We used the National Health Information Database provided by the National Health Insurance Service individually linked to mortality registration data of Statistics Korea between 2008 and 2017. Annual abridged life tables were constructed and Arriaga’s life expectancy decomposition method was employed to estimate age- and cause-specific contributions to the life expectancy gap between National Health Insurance beneficiaries and Medical Aid recipients. The life expectancy difference between National Health Insurance beneficiaries and Medical Aid recipients was 14.5 years during the period of 2008–2017. The age groups between 30 and 64 years accounted for 78.7% and 67.5% of the total life expectancy gap in men and women, respectively. Cancer was the leading cause of death contributing to excess mortality among Medical Aid recipients compared to National Health Insurance beneficiaries. More specifically, alcohol-attributable deaths (such as alcoholic liver disease, liver cancer, liver cirrhosis, and alcohol/substance abuse), suicide, and cardiometabolic risk factor–related deaths (such as cerebrovascular disease, ischemic heart disease, and diabetes) were the leading contributors to the life expectancy gap. To decrease excess deaths in Medical Aid recipients and reduce health inequalities, effective policies for tobacco and alcohol regulation, suicide prevention, and interventions to address cardiometabolic risk factors are needed.

## Introduction

The Republic of Korea (hereafter ‘Korea’) achieved universal health coverage in 1989 by expanding the National Health Insurance program. The government-funded Medical Aid system provides healthcare benefits for low-income families, and its recipients account for around 3% of the total Korean population [1]. Several studies have reported poor health status and higher mortality rates among Medical Aid recipients compared to National Health Insurance beneficiaries in Korea [2–5]. A recent Korean study showed that the life expectancy of Medical Aid recipients was significantly shorter than that of National Health Insurance beneficiaries [6]. However, there is a paucity of information on the contributions of age and causes of death to the life expectancy gap between Medical Aid recipients and National Health Insurance beneficiaries. Quantifying the contribution to life expectancy differences between the two populations by age group and cause of death is important for identifying potential pathways responsible for the life expectancy gap and for developing intervention strategies to reduce excess mortality in the marginalized population. This study aimed to quantify the contribution of age and causes of death to the life expectancy difference between Medical Aid recipients and National Health Insurance beneficiaries during the last 10 years between 2008 and 2017 in Korea based on whole-population data from the National Health Insurance Service (NHIS).

## Materials and methods

### Data and study population

We used the National Health Information Database (NHID) provided by the NHIS. The NHID was linked to mortality registration data from Statistics Korea between 2008 and 2017 [7]. The NHID data cover all Medical Aid recipients and National Health Insurance beneficiaries in Korea except for foreigners. According to the National Health Insurance Act, National Health Insurance beneficiaries are Korean nationals who reside within the country, excluding those who receive government-funded medical benefits. Medical Aid recipients were defined as recipients of government-funded medical benefits on the first day of the year based on the Medical Care Assistance Act. Considering the small numbers of deaths among the 0, 1–4, 5–9, and 10–15 age groups, especially among Medical Aid recipients, 10-year data were combined to calculate life expectancies and to compute age- and cause- specific contributions to the life expectancy difference between Medical Aid recipients and National Health Insurance beneficiaries. The numbers of population and deaths during the study period (2008–2017) according to sex, age groups (0, 1–4, 5–9, 10–14, …, 85+), causes of death, and eligibility (Medical Aid or National Health Insurance) were obtained from the NHID. S1 Table presents the numbers of population and deaths according to eligibility during the study period. Between 2008 and 2017, a total of 502,938,403 subjects (486,985,349 National Health Insurance beneficiaries and 15,953,054 Medical Aid recipients) and 2,584,405 deaths (2,226,516 National Health Insurance beneficiaries and 357,889 Medical Aid recipients) were analyzed. Causes of death were coded according to the International Classification of Diseases 10th Revision (ICD-10) and categorized into 15 broad groups (see S3 Table) and 60 detailed causes (see S4 Table).

### Statistical analysis

Annual abridged life tables were constructed using 5-year probabilities of death separately for Medical Aid recipients and National Health Insurance beneficiaries according to sex. Standard life table procedures were used to calculate the survival rates [8], and the Kannisto-Thatcher method was employed to expand the open-ended age interval of 85+ to estimate the probability of dying for each of the 5-year age groups of 85–89, 90–94, …, 120–124, and 125+ [9]. We employed Arriaga’s life expectancy decomposition method to estimate age- and cause-specific contributions to the life expectancy difference between National Health Insurance beneficiaries and Medical Aid recipients. Arriaga’s method was used to partition the absolute difference in life expectancy between National Health Insurance beneficiaries and Medical Aid recipients into age- and cause- components. The life expectancy difference was first decomposed by age group and then by cause of death within an age group according to their proportion. Age-specific contributions reflect the impact of mortality in the age groups on the life expectancy difference between two populations, and contributions of each cause are the combined effect of each proportionate contribution of causes among all age groups. The detailed analytical procedures, including the mathematical formulae, have been described elsewhere [10–12]. The Kannisto-Thatcher method and Arriaga’s life expectancy decomposition method have been used in previous Korean studies [13–16]. The National Health Insurance Service of Korea and the Seoul National University Hospital Institutional Review Board approved all aspects of this study.

## Results

As shown in Table 1, during the 10-year study period from 2008 to 2017, the life expectancy difference between National Health Insurance beneficiaries and Medical Aid recipients was 14.47 years. The difference was larger in men (17.54 years) than in women (10.24 years). The sex difference in life expectancy was more than twice as great in Medical Aid recipients than in National Health Insurance beneficiaries. The life expectancy difference between men and women was 13.40 years for Medical Aid recipients, while the difference was 6.10 years for National Health Insurance beneficiaries. The life expectancy gap between men with Medical Aid and women with National Health Insurance was 23.64 years (Table 1).

**Table 1:** Life expectancy (years) in National Health Insurance beneficiaries and Medical Aid recipients and life expectancy gap (years) between the two groups in Korea, 2008–2017.

	National Health Insurance	Medical Aid	Difference
**Overall**	82.35	67.88	14.47
**Men**	79.05	61.51	17.54
**Women**	85.15	74.91	10.24
**Sex difference**	6.10	13.40	7.30

**Fig 1:**
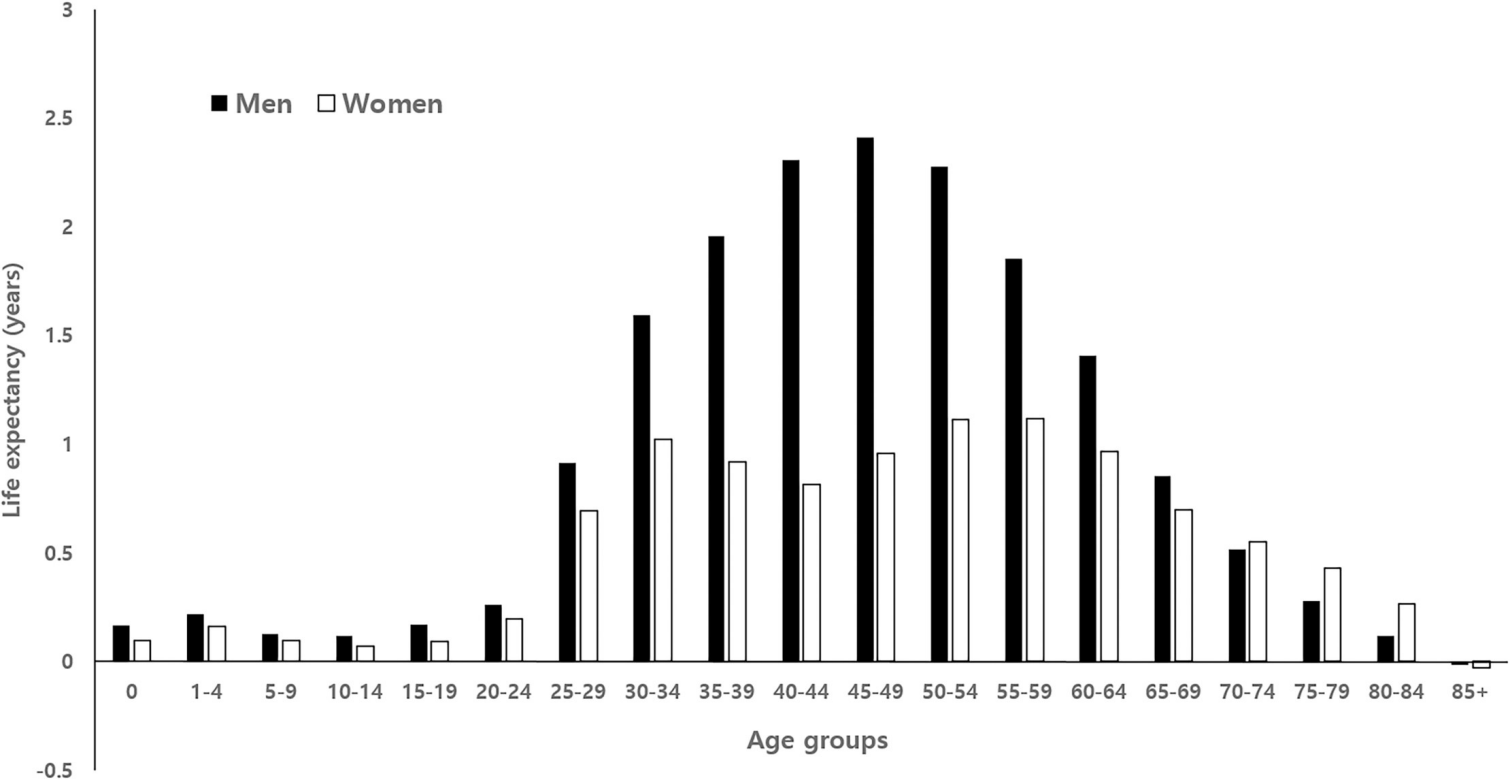
Age-specific contributions to the life expectancy difference between National Health Insurance beneficiaries and Medical Aid recipients in Korea, 2008–2017.

Fig 1 shows that all age groups except for 85+ years positively contributed to the life expectancy difference between National Health Insurance beneficiaries and Medical Aid recipients in both men and women. These positive contributions indicated that the mortality rates in Medical Aid recipients were higher than the mortality rates of National Health Insurance beneficiaries. The middle-adulthood age groups accounted for the major proportion of the age-specific contributions. The age groups between 30 and 64 years accounted for 78.7% and 67.5% of the total life expectancy difference in men and women, respectively (see S2 Table for more detailed values for absolute and percent contributions by age groups).

**Fig 2:**
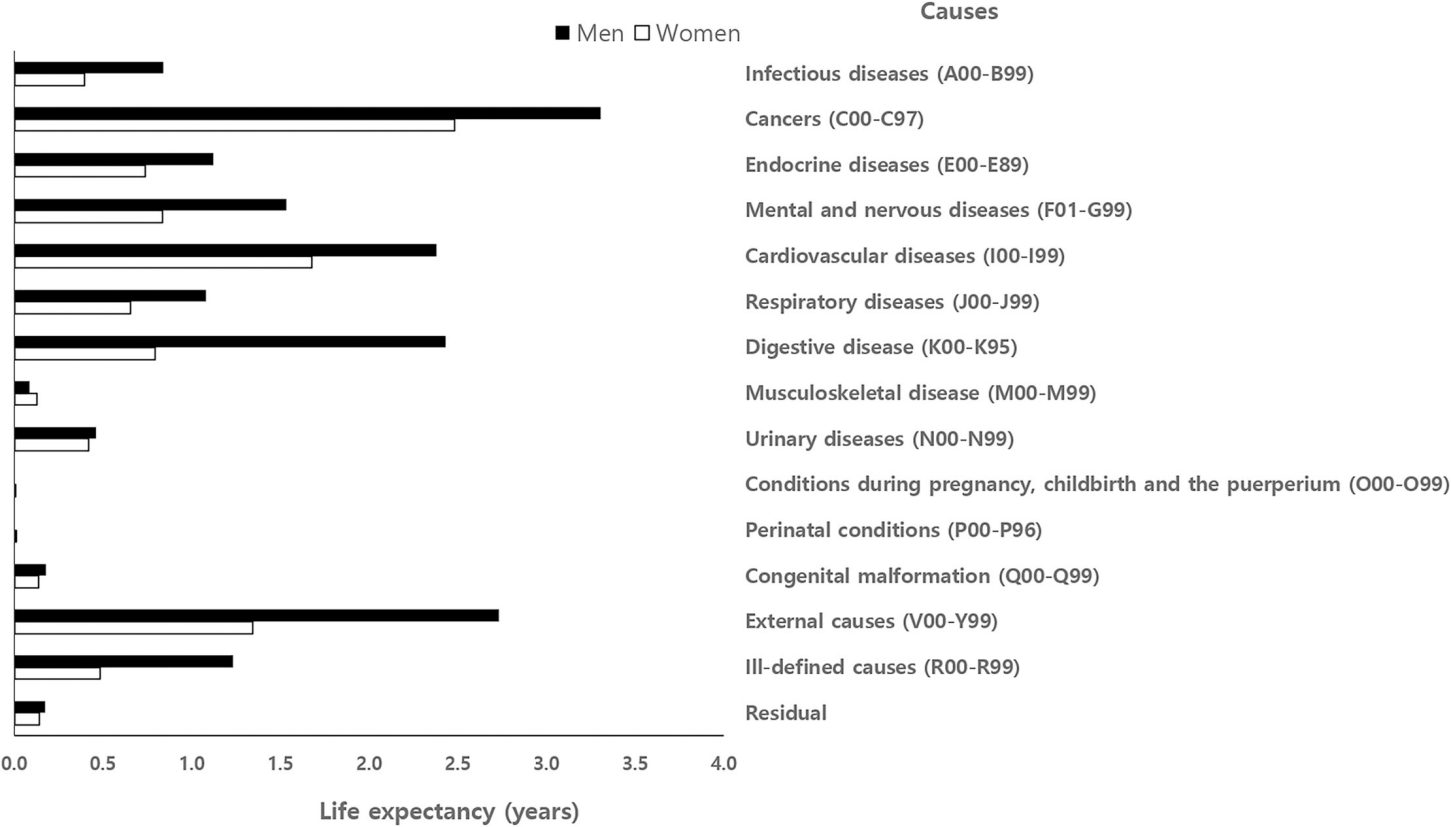
Broad cause-specific contributions to the life expectancy difference between National Health Insurance beneficiaries and Medical Aid recipients in Korea, 2008–2017.

Fig 2 shows cause-specific contributions according to 15 broad causes of death. Cancer was the leading cause of death contributing to the life expectancy gap in both men and women. Cancer, cardiovascular disease, and external causes contributed to the gap by about 50% (48.0% in men and 53.7% in women) of the total life expectancy difference between National Health Insurance beneficiaries and Medical Aid recipients (see S3 Table for more detailed values).

Table 2 presents details on the top 10 causes of death that contributed most to the life expectancy gap between National Health Insurance beneficiaries and Medical Aid recipients by sex. In men and women combined, cerebrovascular diseases(7.0%), suicide (6.9%), alcoholic liver disease (6.9%), diabetes (6.0%), other mental and nervous diseases (5.8%), other cardiovascular diseases (3.7%), liver cancer (3.6%), other external causes (3.4%), pneumonia (3.1%), and ischemic heart disease (2.9%) were the top 10 of the 60 specific causes of death. These 10 causes of death accounted for 49.3% of the total life expectancy difference between National Health Insurance beneficiaries and Medical Aid recipients. When we separately examined the ranks of detailed causes of death contributing to the life expectancy gap by sex, alcoholic liver disease, liver cancer, and liver cirrhosis were more important in men than in women, while cerebrovascular diseases, diabetes, and ischemic heart disease were more important in women than in men. The percent contributions of alcohol-related causes of deaths (alcoholic liver disease, liver cancer, liver cirrhosis, and alcohol/substance abuse) were greater in men (17.3% in total) than in women (7.4% in total). Meanwhile, the percent contributions of causes of death related to cardiometabolic risk factors (such as cerebrovascular disease, ischemic heart disease, and diabetes) were somewhat greater in women (17.2% in total) than in men (15.0% in total) (see S4 Table for more detailed information).

**Table 2:** The top 10 leading causes of death among 60 specific causes contributing to the life expectancy difference between National Health Insurance beneficiaries and Medical Aid recipients according to sex.

Rank	Cause	Years	% contribution
***Men and women combined***			
**1**	**Cerebrovascular diseases (I60-I69)**	1.01	7.0
**2**	**Suicide (X60-X84)**	1.00	6.9
**3**	**Alcoholic liver disease (K70)**	1.00	6.9
**4**	**Diabetes (E10-E14)**	0.87	6.0
**5**	**Other mental and nervous diseases**	0.83	5.8
**6**	**Other cardiovascular diseases**	0.54	3.7
**7**	**Liver cancer (C22)**	0.52	3.6
**8**	**Other external causes**	0.49	3.4
**9**	**Pneumonia (J12-J18)**	0.44	3.1
**10**	**Ischemic heart disease (I20-I25)**	0.42	2.9
	**Sum**	7.12	49.3
***Men***			
**1**	**Alcoholic liver disease (K70)**	1.45	8.3
**2**	**Suicide (X60-X84)**	1.23	7.0
**3**	**Cerebrovascular diseases (I60-I69)**	1.15	6.6
**4**	**Other mental and nervous diseases**	1.01	5.8
**5**	**Diabetes (E10-E14)**	1.00	5.7
**6**	**Liver cancer (C22)**	0.74	4.2
**7**	**Other external causes**	0.65	3.7
**8**	**Other cardiovascular diseases**	0.61	3.5
**9**	**Pneumonia (J12-J18)**	0.56	3.2
**10**	**Other digestive disease**	0.49	2.8
	**Sum**	8.89	50.8
***Women***			
**1**	**Cerebrovascular diseases (I60-I69)**	0.78	7.6
**2**	**Suicide (X60-X84)**	0.73	7.2
**3**	**Diabetes (E10-E14)**	0.65	6.4
**4**	**Other mental and nervous diseases**	0.61	6.0
**5**	**Breast cancer (C50)**	0.46	4.5
**6**	**Other cardiovascular diseases**	0.44	4.3
**7**	**Pneumonia (J12-J18)**	0.34	3.3
**8**	**Ischemic heart disease (I20-I25)**	0.34	3.3
**9**	**Alcoholic liver disease (K70)**	0.33	3.3
**10**	**Chronic renal failure (N18)**	0.32	3.1
	**Sum**	5.00	48.8

## Discussion

During the last 10 years, from 2008 to 2017, the life expectancy difference in Korea between National Health Insurance beneficiaries and Medical Aid recipients was 17.54 years for men and 10.24 years for women. Age-specific decomposition showed that the age groups between 30 and 64 years accounted for 77.0% of the total life expectancy difference. A prior Korean study on the decomposition of life expectancy inequalities by income groups showed that most of the life expectancy gaps between top and bottom income quintiles were due to excess mortality among middle-aged and older adults in the lowest income quintile [17]. The results of this study indicate that excess mortality rates among younger Medical Aid recipients contributed to the life expectancy gap to a greater extent than was found for the bottom income quintile in the previous study [16].

The results of the cause-specific decomposition analysis showed that, among 15 broad causes of death, cancer, cardiovascular disease, external causes, and digestive disease were the major factors contributing to excess mortality among Medical Aid recipients compared to National Health Insurance beneficiaries. Cancer was the leading cause of death, contributing to 18.8% of the life expectancy gap in men and to 24.2% of the life expectancy gap in women. The excess mortality from cancer deaths among male Medical Aid recipients mostly occurred in the middle-aged population, aged between 40 and 59 years (see S1 Fig). The percent contributions of lung cancer and chronic lower respiratory disease were found to be similar in men and women (S4 Table). Prior studies showed that cardiovascular disease, rather than cancer, was the most important cause of death among Korean women and explained the largest proportion of inequalities in mortality and life expectancy [17, 18]. The results of this study suggest that exposure to cigarette smoking and other carcinogens may be major contributors to the lower life expectancy among Medical Aid recipients. Several studies have revealed that a greater smoking prevalence was observed in both Korean men and women with low socioeconomic position [19, 20]. However, smoking prevalence, especially among female Medical Aid recipients, has not been reported due to issues related to the small sample size of survey participants covered by the Medical Aid program. A future study is needed to examine smoking prevalence among Medical Aid recipients in Korea.

Our further analysis of 60 detailed causes of death indicated that alcohol-attributable deaths, suicide, and cerebrovascular diseases were the leading contributors to the life expectancy gap between the two groups. Alcohol-related causes of death, such as alcoholic liver disease, liver cancer, liver cirrhosis, and alcohol/substance abuse, made significant contributions to the life expectancy gap, especially in men. These alcohol-related causes of death accounted for 3.03 years of the life expectancy difference, or 17.3% of the total difference between Medical Aid recipients and National Health Insurance beneficiaries among men. The excess mortality from alcohol-related deaths among male Medical Aid recipients mostly occurred in the middle-age population between 35 and 59 years of age (see S2 Fig). Increased risks of alcohol-attributable mortality among socially disadvantaged men have been found in several studies [21–24]. In addition, alcohol-related deaths also made substantial contributions to sex differences in life expectancy [25]. Meanwhile, the contribution of alcohol-related deaths (i.e., alcoholic liver disease, liver cancer, liver cirrhosis, and alcohol/substance abuse) was only 0.75 years in women. In Korea, after the economic crisis in the late 1990s, socioeconomic inequalities in alcohol-attributable deaths among Korean men aged 40–59 years increased [26]. According to the Global Burden of Disease Study 2016, alcohol accounted for 6.7% percent of total attributable deaths among Korean men in 2016, in contrast to 2.2% for women [27] The findings of this study suggest that alcohol regulation policies are urgently necessary to reduce premature deaths in the most deprived men. Alcohol regulation policies specifically targeting socially marginalized men would be effective in reducing both health inequalities and the sex gap in life expectancy.

Suicide, the second leading cause of death contributing to the gap in life expectancy between Medical Aid recipients and National Health Insurance beneficiaries, was more prevalent in working-age Medical Aid recipients (especially those aged 25–54 years) for both men and women (see S3 Fig). Suicide accounted for 6.9% of the total life expectancy difference (1.23 years for men and 0.73 years for women) between National Health Insurance beneficiaries and Medical Aid recipients in both men and women. A prior Korean study showed that Medical Aid recipients had a higher suicide risk than high-income National Health Insurance beneficiaries between 2003 and 2013 [4]. Suicide has been a major cause of death in Korea for several decades. In addition, the suicide rates of middle-aged men (30–49 years) showed a continuously increasing trend between 1993 and 2006, while the suicide rate among women has decreased since 2010 [28] The results of this study showed that the age-specific contribution of suicide to the life expectancy difference peaked at 30–39 years of age in both men and women. This finding implies that specific suicide prevention policies for socially marginalized early middle-aged adults should be implemented in addition to current policies focused on adolescents and the elderly. The excess deaths due to suicide in Medical Aid recipients compared to National Health Insurance beneficiaries might be due to limited access to mental health professionals. Alternatively, stressful situations triggered by economic hardship might lead individuals to commit suicide, given that excess suicide deaths mainly occurred in the working-age population. The link between alcohol and suicide might also explain the high contribution of suicide to the life expectancy difference between Medical Aid recipients and National Health Insurance beneficiaries [29].

In women, causes of death such as cerebrovascular disease, ischemic heart disease, and diabetes, which are closely related to cardiometabolic risk factors (hypertension, blood glucose, blood lipid profile, and high body mass index), were somewhat more important than in men. Clear educational inequalities in high blood pressure, abdominal obesity, and blood lipid profiles have been reported in Korean women in various age cohorts, while in men this pattern of inequality has only emerged in younger age cohorts [30]. Unlike socioeconomic inequalities in obesity measures in Korean men, a clear pattern of socioeconomic inequality in obesity measures has been reported among Korean women [31]. Active interventions to address cardiometabolic risk factors might be helpful to attenuate the life expectancy gap between Medical Aid recipients and National Health Insurance beneficiaries, especially among Korean women.

This study has strengths and limitations. This study used whole-population data during a 10-year period with individual linkage between NHID and death registers in Korea. So far as we are aware, this is the first study to explore age-and cause specific contributions to the life expectancy difference between Medical Aid recipients and National Health Insurance beneficiaries. However, this age- and cause-specific decomposition analysis only provides clues about the mechanisms responsible for the life expectancy gap between Medical Aid recipients and National Health Insurance beneficiaries. Further studies should examine differences in exposure to risk factors over the life course between National Health Insurance beneficiaries and Medical Aid recipients. It should be noted that poverty rates among the elderly population aged 60 or older increased substantially during recent decades in Korea [32] and the poor elderly are more likely to be Medical Aids recipients [1]. This indicates that health policy programs and interventions for targeting the poor elderly need to be extended.

## Conclusions

In conclusion, this study showed that the lower life expectancy among Medical Aid recipients was mainly attributable to premature deaths in the age groups between 30 and 64 years, alcohol-related deaths, and suicide. Causes of death related to smoking and cardiometabolic risk factors were also important. The government should implement more effective policies for tobacco and alcohol regulation, suicide prevention, and interventions addressing cardiometabolic risk factors to protect the health of Medical Aid beneficiaries, to reduce excess deaths in Medical Aid recipients, and subsequently to reduce health inequalities in Korea.

## Supporting information

S1 Fig The age-specific contribution of cancer to the life expectancy difference between National Health Insurance beneficiaries and Medical Aid recipients between 2008 and 2017 by sex. (PDF)

S2 Fig The age-specific contribution of alcohol-related deaths to the life expectancy difference between National Health Insurance beneficiaries and Medical Aid recipients between 2008 and 2017 among men. (PDF)

S3 Fig The age-specific contribution of suicide to the life expectancy difference between National Health Insurance beneficiaries and Medical Aid recipients between 2008 and 2017 by sex. (PDF)

S1 Table Number of population and deaths during the 10-year study period between 2008 and 2017. (DOCX)

S2 Table Age-specific contributions to the life expectancy difference between National Health Insurance beneficiaries and Medical Aid recipients between 2008 and 2017 by sex. (DOCX)

S3 Table Cause-specific contributions to the life expectancy difference between Medical Aid recipients and National Health Insurance beneficiaries by sex in 2008–2017 combined. (DOCX)

S4 Table Cause-specific contributions to the life expectancy difference according to 60 causes of death. (DOCX)
