# Supplementary material for: Age- and cause-specific contributions to the life expectancy gap between Medical Aid recipients and National Health Insurance beneficiaries in Korea, 2008–2017
Source: PLoS One. 2020 Nov 3;15(11):e0241755. doi: 10.1371/journal.pone.0241755 (PMC7608888; doi:10.1371/journal.pone.0241755)
Supplement: S2 Fig — (PDF) [file pone.0241755.s002.pdf]

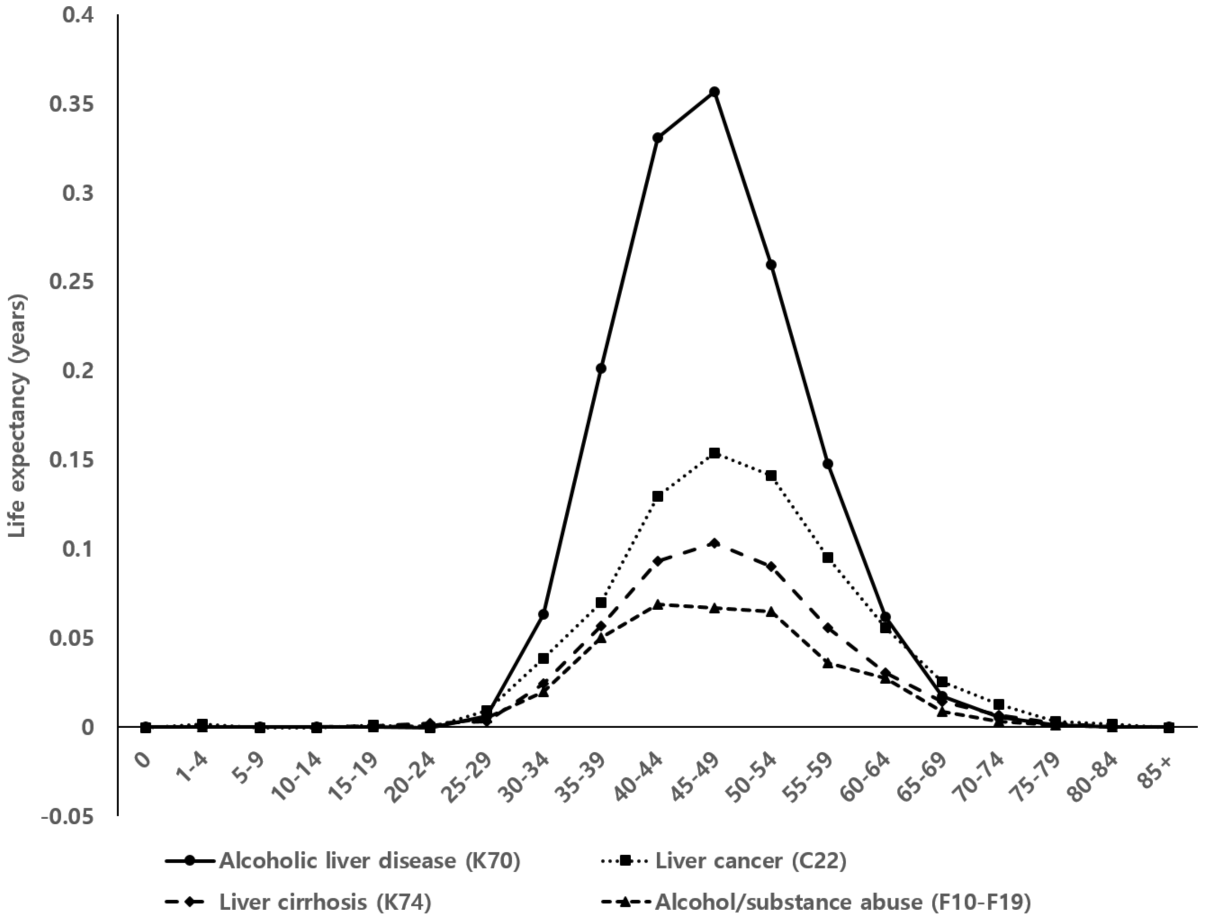

S2 Fig. The age-specific contribution of alcohol-related deaths to the life expectancy difference between National Health Insurance beneficiaries and Medical Aid recipients between 2008 and 2017 among men.
