# Supplementary material for: Age- and cause-specific contributions to the life expectancy gap between Medical Aid recipients and National Health Insurance beneficiaries in Korea, 2008–2017
Source: PLoS One. 2020 Nov 3;15(11):e0241755. doi: 10.1371/journal.pone.0241755 (PMC7608888; doi:10.1371/journal.pone.0241755)
Supplement: S1 Table — (DOCX) [file pone.0241755.s004.docx]

S1 Table. Number of population and deaths during the 10-year study period between 2008 and 2017.

| Year | Overall | | National Health Insurance | | Medical Aid | |
| --- | --- | --- | --- | --- | --- | --- |
|  | No. of population | No. of deaths | No. of population | No. of deaths | No. of population | No. of deaths |
| Overall (men and women) | | | | | | |
| 2008 | 49,245,374 | 239,798 | 47,403,267 | 200,905 | 1,842,107 | 38,893 |
| 2009 | 49,519,213 | 240,935 | 47,689,560 | 204,211 | 1,829,653 | 36,724 |
| 2010 | 49,756,545 | 249,930 | 48,093,925 | 214,021 | 1,662,620 | 35,909 |
| 2011 | 49,980,840 | 252,191 | 48,324,794 | 215,822 | 1,656,046 | 36,369 |
| 2012 | 50,228,255 | 262,277 | 48,634,986 | 225,154 | 1,593,269 | 37,123 |
| 2013 | 50,455,808 | 259,431 | 48,961,585 | 225,323 | 1,494,223 | 34,108 |
| 2014 | 50,658,369 | 262,816 | 49,212,587 | 227,845 | 1,445,782 | 34,971 |
| 2015 | 50,855,927 | 269,976 | 49,437,004 | 235,119 | 1,418,923 | 34,857 |
| 2016 | 51,042,438 | 271,023 | 49,524,158 | 236,893 | 1,518,280 | 34,130 |
| 2017 | 51,195,634 | 276,028 | 49,703,483 | 241,223 | 1,492,151 | 34,805 |
| Sum | 502,938,403 | 2,584,405 | 486,985,349 | 2,226,516 | 15,953,054 | 357,889 |
| Men | | | | | | |
| 2008 | 24,681,837 | 132,347 | 23,876,217 | 113,179 | 805,620 | 19,168 |
| 2009 | 24,814,033 | 133,287 | 24,011,827 | 115,339 | 802,206 | 17,948 |
| 2010 | 24,922,943 | 138,129 | 24,195,480 | 120,658 | 727,463 | 17,471 |
| 2011 | 25,023,245 | 139,216 | 24,299,816 | 121,852 | 723,429 | 17,364 |
| 2012 | 25,136,066 | 143,509 | 24,436,925 | 125,925 | 699,141 | 17,584 |
| 2013 | 25,237,439 | 141,885 | 24,582,055 | 125,684 | 655,384 | 16,201 |
| 2014 | 25,327,681 | 143,549 | 24,692,284 | 126,767 | 635,397 | 16,782 |
| 2015 | 25,415,837 | 146,065 | 24,786,628 | 129,277 | 629,209 | 16,788 |
| 2016 | 25,498,033 | 146,293 | 24,820,487 | 129,393 | 677,546 | 16,900 |
| 2017 | 25,563,384 | 148,139 | 24,893,770 | 131,104 | 669,614 | 17,035 |
| Sum | 251,620,498 | 1,412,419 | 244,595,489 | 1,239,178 | 7,025,009 | 173,241 |
| Women | | | | | | |
| 2008 | 24,563,537 | 107,451 | 23,527,050 | 87,726 | 1,036,487 | 19,725 |
| 2009 | 24,705,180 | 107,648 | 23,677,733 | 88,872 | 1,027,447 | 18,776 |
| 2010 | 24,833,602 | 111,801 | 23,898,445 | 93,363 | 935,157 | 18,438 |
| 2011 | 24,957,595 | 112,975 | 24,024,978 | 93,970 | 932,617 | 19,005 |
| 2012 | 25,092,189 | 118,768 | 24,198,061 | 99,229 | 894,128 | 19,539 |
| 2013 | 25,218,369 | 117,546 | 24,379,530 | 99,639 | 838,839 | 17,907 |
| 2014 | 25,330,688 | 119,267 | 24,520,303 | 101,078 | 810,385 | 18,189 |
| 2015 | 25,440,090 | 123,911 | 24,650,376 | 105,842 | 789,714 | 18,069 |
| 2016 | 25,544,405 | 124,730 | 24,703,671 | 107,500 | 840,734 | 17,230 |
| 2017 | 25,632,250 | 127,889 | 24,809,713 | 110,119 | 822,537 | 17,770 |
| Sum | 251,317,905 | 1,171,986 | 242,389,860 | 987,338 | 8,928,045 | 184,648 |
