# Supplementary material for: Age- and cause-specific contributions to the life expectancy gap between Medical Aid recipients and National Health Insurance beneficiaries in Korea, 2008–2017
Source: PLoS One. 2020 Nov 3;15(11):e0241755. doi: 10.1371/journal.pone.0241755 (PMC7608888; doi:10.1371/journal.pone.0241755)
Supplement: S2 Table — (DOCX) [file pone.0241755.s005.docx]

S2 Table. Age-specific contributions to the life expectancy difference between National Health Insurance beneficiaries and Medical Aid recipients between 2008 and 2017 by sex

|  | Overall | | Men | | Women | |
| --- | --- | --- | --- | --- | --- | --- |
| Age | Years | % Contribution | Years | % Contribution | Years | % Contribution |
| 0 | 0.13 | 0.9 | 0.17 | 1.0 | 0.10 | 0.9 |
| 1-4 | 0.19 | 1.3 | 0.22 | 1.3 | 0.16 | 1.6 |
| 5-9 | 0.11 | 0.8 | 0.13 | 0.7 | 0.10 | 0.9 |
| 10-14 | 0.10 | 0.7 | 0.12 | 0.7 | 0.07 | 0.7 |
| 15-19 | 0.14 | 0.9 | 0.17 | 1.0 | 0.09 | 0.9 |
| 20-24 | 0.23 | 1.6 | 0.26 | 1.5 | 0.20 | 1.9 |
| 25-29 | 0.83 | 5.7 | 0.91 | 5.2 | 0.69 | 6.8 |
| 30-34 | 1.29 | 8.9 | 1.59 | 9.1 | 1.02 | 10.0 |
| 35-39 | 1.37 | 9.5 | 1.96 | 11.2 | 0.92 | 9.0 |
| 40-44 | 1.52 | 10.5 | 2.31 | 13.2 | 0.81 | 8.0 |
| 45-49 | 1.85 | 12.8 | 2.41 | 13.8 | 0.96 | 9.3 |
| 50-54 | 2.03 | 14.0 | 2.28 | 13.0 | 1.12 | 10.9 |
| 55-59 | 1.77 | 12.2 | 1.86 | 10.6 | 1.12 | 10.9 |
| 60-64 | 1.32 | 9.1 | 1.41 | 8.0 | 0.97 | 9.4 |
| 65-69 | 0.77 | 5.3 | 0.85 | 4.9 | 0.70 | 6.8 |
| 70-74 | 0.47 | 3.3 | 0.52 | 2.9 | 0.55 | 5.4 |
| 75-79 | 0.29 | 2.0 | 0.28 | 1.6 | 0.43 | 4.2 |
| 80-84 | 0.15 | 1.0 | 0.12 | 0.7 | 0.27 | 2.6 |
| 85+ | -0.08 | -0.6 | -0.01 | -0.1 | -0.03 | -0.3 |
| Total | 14.47 | 100.0 | 17.55 | 100.0 | 10.24 | 100.0 |
