# Supplementary material for: Age- and cause-specific contributions to the life expectancy gap between Medical Aid recipients and National Health Insurance beneficiaries in Korea, 2008–2017
Source: PLoS One. 2020 Nov 3;15(11):e0241755. doi: 10.1371/journal.pone.0241755 (PMC7608888; doi:10.1371/journal.pone.0241755)
Supplement: S3 Table — (DOCX) [file pone.0241755.s006.docx]

S3 Table. Cause-specific contributions to the life expectancy difference between Medical Aid recipients and National Health Insurance beneficiaries by sex in 2008-2017 combined

| Causes of death | Overall | | Men | | Women | |
| --- | --- | --- | --- | --- | --- | --- |
|  | Years | % Contribution | Years | % Contribution | Years | % Contribution |
| Infectious diseases (A00-B99) | 0.64 | 4.4 | 0.84 | 4.8 | 0.39 | 3.8 |
| Cancers (C00-C97) | 2.96 | 20.5 | 3.30 | 18.8 | 2.48 | 24.2 |
| Endocrine diseases (E00-E89) | 0.98 | 6.8 | 1.12 | 6.4 | 0.74 | 7.2 |
| Mental and nervous diseases (F01-G99) | 1.22 | 8.4 | 1.53 | 8.7 | 0.83 | 8.2 |
| Cardiovascular diseases (I00-I99) | 2.09 | 14.4 | 2.38 | 13.6 | 1.68 | 16.4 |
| Respiratory diseases (J00-J99) | 0.85 | 5.9 | 1.08 | 6.2 | 0.66 | 6.4 |
| Digestive disease (K00-K95) | 1.76 | 12.2 | 2.43 | 13.8 | 0.79 | 7.8 |
| Musculoskeletal disease (M00-M99) | 0.10 | 0.7 | 0.09 | 0.5 | 0.13 | 1.2 |
| Urinary diseases (N00-N99) | 0.44 | 3.0 | 0.46 | 2.6 | 0.42 | 4.1 |
| Conditions during pregnancy, childbirth and the puerperium (O00-O99) | 0.00 | 0.0 | 0.00 | 0.0 | 0.01 | 0.1 |
| Perinatal conditions (P00-P96) | 0.00 | 0.0 | 0.00 | 0.0 | 0.01 | 0.1 |
| Congenital malformation (Q00-Q99) | 0.16 | 1.1 | 0.18 | 1.0 | 0.14 | 1.3 |
| External causes (V00-Y99) | 2.11 | 14.6 | 2.73 | 15.6 | 1.34 | 13.1 |
| Ill-defined causes (R00-R99) | 0.98 | 6.8 | 1.23 | 7.0 | 0.48 | 4.7 |
| Residual | 0.16 | 1.1 | 0.17 | 1.0 | 0.14 | 1.4 |
| Total | 14.47 | 100.0 | 17.54 | 100.0 | 10.24 | 100.0 |
