# Supplementary material for: Age- and cause-specific contributions to the life expectancy gap between Medical Aid recipients and National Health Insurance beneficiaries in Korea, 2008–2017
Source: PLoS One. 2020 Nov 3;15(11):e0241755. doi: 10.1371/journal.pone.0241755 (PMC7608888; doi:10.1371/journal.pone.0241755)
Supplement: S4 Table — (DOCX) [file pone.0241755.s007.docx]

S4 Table. Cause-specific contributions to the life expectancy difference according to 60 causes of death

|  | Overall | | Men | | Women | |
| --- | --- | --- | --- | --- | --- | --- |
| Causes | Years | % Contribution | Years | % Contribution | Years | % Contribution |
| Tuberculosis (A15-A19) | 0.236 | 1.63 | 0.335 | 1.91 | 0.106 | 1.03 |
| Septicemia (A40-A41) | 0.137 | 0.95 | 0.157 | 0.89 | 0.116 | 1.13 |
| Other infectious diseases | 0.267 | 1.85 | 0.346 | 1.97 | 0.173 | 1.69 |
| Esophageal cancer (C15) | 0.060 | 0.41 | 0.087 | 0.50 | 0.017 | 0.16 |
| Stomach cancer (C16) | 0.369 | 2.55 | 0.451 | 2.57 | 0.238 | 2.32 |
| Colorectal cancer (C18) | 0.152 | 1.05 | 0.169 | 0.96 | 0.128 | 1.25 |
| Rectal cancer (C19-C21) | 0.174 | 1.20 | 0.204 | 1.16 | 0.123 | 1.20 |
| **Liver cancer (C22)** | 0.520 | **3.60** | 0.739 | **4.21** | 0.207 | **2.02** |
| Biliary tract/gall bladder cancer (C23-24) | 0.060 | 0.41 | 0.061 | 0.35 | 0.058 | 0.57 |
| Pancreatic cancer (C25) | 0.076 | 0.52 | 0.092 | 0.52 | 0.056 | 0.55 |
| Lung and bronchus cancer (C33-C34) | 0.354 | 2.44 | 0.456 | 2.60 | 0.260 | 2.54 |
| Breast cancer (C50) | 0.204 | 1.41 | 0.002 | 0.01 | 0.462 | 4.51 |
| Uterine cervix cancer (C53) | 0.114 | 0.79 | 0.000 | 0.00 | 0.249 | 2.43 |
| Other and unspecified uterine cancer (C54-C55) | 0.013 | 0.09 | 0.000 | 0.00 | 0.030 | 0.29 |
| Ovarian cancer (C56) | 0.046 | 0.32 | 0.000 | 0.00 | 0.106 | 1.04 |
| Prostatic cancer (C61) | 0.009 | 0.06 | 0.018 | 0.10 | 0.000 | 0.00 |
| Kidney cancer (C64) | 0.033 | 0.23 | 0.042 | 0.24 | 0.022 | 0.21 |
| Urinary bladder cancer (C67) | 0.027 | 0.19 | 0.041 | 0.23 | 0.010 | 0.10 |
| Brain and CNS cancer (C70-C72) | 0.131 | 0.90 | 0.157 | 0.89 | 0.104 | 1.02 |
| Thyroid cancer (C73) | 0.007 | 0.05 | 0.006 | 0.03 | 0.009 | 0.09 |
| Non-Hodgkin's lymphoma (C82-C86) | 0.063 | 0.44 | 0.083 | 0.47 | 0.044 | 0.43 |
| Multiple myeloma (C90) | 0.019 | 0.13 | 0.020 | 0.12 | 0.015 | 0.15 |
| Leukemia (C91-C95) | 0.190 | 1.32 | 0.240 | 1.37 | 0.136 | 1.33 |
| Other cancers | 0.340 | 2.35 | 0.436 | 2.48 | 0.206 | 2.01 |
| **Diabetes (E10-E14)** | 0.875 | **6.05** | 1.000 | **5.70** | 0.653 | **6.38** |
| Other endocrine diseases | 0.107 | 0.74 | 0.122 | 0.69 | 0.084 | 0.82 |
| Dementia (F01-F09, G30) | 0.102 | 0.71 | 0.117 | 0.67 | 0.116 | 1.13 |
| Meningitis (G00, G03) | 0.006 | 0.04 | 0.006 | 0.03 | 0.006 | 0.06 |
| **Alcohol/substance abuse (F10-F19)** | 0.230 | **1.59** | 0.353 | **2.01** | 0.055 | **0.53** |
| Parkinson's disease (G20) | 0.047 | 0.33 | 0.047 | 0.27 | 0.046 | 0.45 |
| Other mental and nervous diseases | 0.833 | 5.76 | 1.010 | 5.76 | 0.612 | 5.97 |
| Rheumatic heart disease (I00-I09) | 0.014 | 0.10 | 0.012 | 0.07 | 0.016 | 0.16 |
| Hypertensive disease (I10-I13) | 0.104 | 0.72 | 0.119 | 0.68 | 0.111 | 1.08 |
| **Ischemic heart disease (I20-I25)** | 0.420 | **2.90** | 0.481 | **2.74** | 0.335 | **3.27** |
| **Cerebrovascular accidents (I60-I69)** | 1.012 | **6.99** | 1.153 | **6.57** | 0.776 | **7.58** |
| Other cardiovascular diseases | 0.538 | 3.72 | 0.613 | 3.49 | 0.437 | 4.27 |
| Influenza (J09-J11) | 0.009 | 0.06 | 0.010 | 0.06 | 0.008 | 0.07 |
| Pneumonia (J12-J18) | 0.443 | 3.06 | 0.562 | 3.20 | 0.337 | 3.29 |
| Chronic lower respiratory disease (J40-J47) | 0.227 | 1.57 | 0.294 | 1.67 | 0.175 | 1.71 |
| Other respiratory disease | 0.175 | 1.21 | 0.215 | 1.22 | 0.136 | 1.33 |
| **Alcoholic liver disease (K70)** | 0.997 | **6.89** | 1.454 | **8.28** | 0.333 | **3.25** |
| **Liver cirrhosis (K74)** | 0.354 | **2.44** | 0.483 | **2.75** | 0.159 | **1.55** |
| Other digestive disease | 0.411 | 2.84 | 0.492 | 2.81 | 0.302 | 2.95 |
| Musculoskeletal disease (M00-M99) | 0.103 | 0.71 | 0.087 | 0.50 | 0.126 | 1.23 |
| Chronic renal failure (N18) | 0.338 | 2.34 | 0.351 | 2.00 | 0.318 | 3.10 |
| Other urinary disease | 0.102 | 0.70 | 0.111 | 0.63 | 0.099 | 0.97 |
| Conditions during pregnancy, childbirth and the puerperium (O00-O99) | 0.004 | 0.03 | 0.000 | 0.00 | 0.007 | 0.07 |
| Perinatal conditions (P00-P96) | 0.005 | 0.03 | 0.000 | 0.00 | 0.011 | 0.10 |
| Congenital malformation (Q00-Q99) | 0.158 | 1.09 | 0.176 | 1.00 | 0.135 | 1.32 |
| Transport accidents (V01-V99) | 0.264 | 1.83 | 0.388 | 2.21 | 0.121 | 1.18 |
| Falls (W00-W19) | 0.144 | 0.99 | 0.203 | 1.16 | 0.063 | 0.62 |
| Drowning (W65-W74) | 0.050 | 0.35 | 0.069 | 0.39 | 0.027 | 0.26 |
| Exposure to fire (X00-X09) | 0.037 | 0.26 | 0.047 | 0.27 | 0.021 | 0.21 |
| Accidental poisoning (X40-X49) | 0.023 | 0.16 | 0.034 | 0.20 | 0.012 | 0.11 |
| **Suicide (X60-X84)** | 1.003 | **6.93** | 1.228 | **7.00** | 0.733 | **7.15** |
| Homicide (X85-Y09) | 0.104 | 0.72 | 0.108 | 0.62 | 0.095 | 0.93 |
| Other external causes | 0.486 | 3.36 | 0.654 | 3.73 | 0.273 | 2.67 |
| Senility (R54) | 0.083 | 0.57 | 0.035 | 0.20 | 0.036 | 0.35 |
| Other ill-defined causes | 0.901 | 6.23 | 1.197 | 6.82 | 0.448 | 4.37 |
| Residual | 0.157 | 1.09 | 0.172 | 0.98 | 0.141 | 1.38 |
| Total | 14.467 | 100.0 | 17.545 | 100.0 | 10.240 | 100.0 |
